# *Carthamus tinctorius* L. Seed and *Taraxacum coreanum* Attenuate Oxidative Stress Induced by Hydrogen Peroxide in SH-SY5Y Cells

**DOI:** 10.3390/foods12193617

**Published:** 2023-09-28

**Authors:** Mei Tong He, Chan Hum Park, Yu Su Shin, Ji Hyun Kim, Eun Ju Cho

**Affiliations:** 1 College of Korean Medicine, Gachon University, Seongnam 13120, Republic of Korea; ellenho@gachon.ac.kr; 2 Institute of New Frontier Research Team, Research Institute of Medical-Bio Convergence, Hallym University, Chuncheon 24252, Republic of Korea; ptman123@naver.com; 3 Department of Medicinal Crop Research, National Institute of Horticultural and Herbal Science, Rural Development Administration, Eumseong 27709, Republic of Korea; totoro69@korea.kr; 4 Department of Food Science and Nutrition, Gyeongsang National University, Jinju 52725, Republic of Korea; 5 Department of Food Science and Nutrition and Kimchi Research Institute, Pusan National University, Busan 46241, Republic of Korea

**Keywords:** *Carthamus tinctorius* L. seed, free radicals, herbal medicines, oxidative stress, synergy, *Taraxacum coreanum*

## Abstract

Oxidative stress is closely associated with the pathology of neurodegenerative diseases. The seeds of *Carthamus tinctorius* L. (CTS) and *Taraxacum coreanum* (TC) are reported as herbal medicines for neuroprotection. This study investigated the protective effect of CTS, TC, and their combination against oxidative stress induced by H_2_O_2_ in SH-SY5Y cells. The CTS and TC combination dose-dependently increased DPPH and ·OH radical scavenging activities compared with non-combination. The combination showed a higher increased cell survival rate in H_2_O_2_-stimulated SH-SY5Y cells than CTS or TC. Moreover, CTS, TC, and their combination-treated cells reduced LDH release and apoptotic cells. CTS, TC, and their combination also inhibited NO and ROS generation. Further, the combination of up-regulated antioxidant enzymes (superoxide dismutase and glutathione peroxidase) and Bcl-2 protein expressions and down-regulated Bax expression. These findings suggest that the combination of CTS and TC may be beneficial to prevent and treat oxidative stress-mediated neurodegenerative diseases.

## 1. Introduction

Oxidative stress occurs when the pro-oxidants and antioxidants are imbalanced, which is defined as a growth of reactive oxygen/nitrogen species (ROS/RNS) or a loss of antioxidants in living cells [1]. The overproduction of free radicals results in an increase in ROS/RNS, simultaneously evoking mitochondrial dysfunction and oxidative DNA damage, resulting in an increase in apoptotic cells [2,3]. It is important that the increase in ROS/RNS in the neuronal cells is involved in many neurodegenerative diseases including Alzheimer’s disease, Parkinson’s disease, and Huntington’s disease [4]. Therefore, oxidative stress is a feature related to neuron degeneration and neuronal cell death.

Herbal medicines, which contain various phytochemicals such as polyphenols, vitamins, and flavonoids have been widely used as natural antioxidants to inhibit oxidative stress [5]. The seed of *Carthamus tinctorius* L. (CTS) has been used for studying pharmacological activities, such as anti-inflammation, antioxidation, and anti-diabetes [6,7]. *Taraxacum coreanum* (TC) has been used for the treatment of inflammatory diseases, such as rheumatic disease and gastritis, for a long time [8,9,10]. Several studies reported the neuroprotective effects of CTS and TC, respectively. The CTS attenuated the oxidative damage by suppressing lipid peroxidation and nitric oxide (NO) production in chronic alcohol-induced mouse brain and C6 glial cells [11,12]. TC is protected from neurotoxicity by regulating heme oxygenase-1 (HO-1)/nuclear factor erythroid 2-related factor 2 pathway in mouse hippocampal HT22 cells [13]. However, the CTS and TC combination on neuroprotective effects in oxidative stress-induced human neuroblastoma SH-SY5Y cells has not been studied.

In many oxidative stress-related diseases, combination therapy instead of monotherapy is applied to provide better therapeutic effects (synergy effects) [14,15]. Our previous study has demonstrated that an equal amount of CTS and TC mixture attenuated amyloid beta-induced cognitive dysfunction in mice by enhancing memory function and down-regulating the amyloidogenic pathway [16]. However, the protective effects of the combination with CTS and TC against H_2_O_2_-stimulated neuronal oxidative damage in SH-SY5Y cells are still unclear. Herein, we investigated whether the CTS and TC combination at a ratio of 1:1 has synergistic free radical scavenging activities and protective effects of the combination against H_2_O_2_-stimulated oxidative stress in the SH-SY5Y human neuroblastoma cell line.

## 2. Materials and Methods

### 2.1. Materials

#### 2.1.1. Sample Preparation

Bulleted lists are as follows. Water extracts of CTS, TC, and the combination (CTS + TC) were contributed by the National Institute of Horticultural and Herbal Science, Rural Development Administration (Eumseong, Republic of Korea). Dried and ground CTS, TC, and CTS + TC (1:1) were placed into conical flasks independently. Water was added, and the contents were stirred and extracted for 8 h. Water extracts were then concentrated and freezer-dried for 48 h [17]. The yields of CTS, TC, and CTS + TC were 8.4%, 30.6%, and 26.3%, respectively. Sample stocks were dissolved in DMSO for use.

#### 2.1.2. Reagents

The following reagents were purchased from Sigma-Aldrich (St. Louis, MO, USA): 1,1-Dephenyl-2-picrylhydrazyl (DPPH), 2′,7′-dichlorofluorescein diacetate (DCF-DA), 2-deoxyribose, and Griess reagent. Dulbecco’s modified eagle medium (DMEM), fetal bovine serum (FBS), and penicillin-streptomycin were acquired from Welgene (Daegu, Republic of Korea). Bio Basic Inc. (Toronto, Canada) provided the 3-(4,5-Dimethylthiazol-2-yl)-2,3-diphenyl tetrazolium bromide (MTT). Dimethyl sulfoxide (DMSO) and FeSO_4_·7H_2_O were obtained from Daejung Chemicals & Metals Co. Ltd. (Gyeonggi-do, Republic of Korea). H_2_O_2_ was purchased from Junsei (Tokyo, Japan). Thiobarbituric acid (TBA) and trichloroacetic acid (TCA) were bought from Acros Organics (Morris Plains, NJ, USA) and Kanto Chemical Co., Inc. (Tokyo, Japan), respectively. The radioimmunoprecipitation assay (RIPA) buffer was received from Elpics Biotech (Daejeon, Republic of Korea), the protease inhibitor cocktail was purchased from Calbiochem (Cambridge, MA, USA), and the polyvinylidenefluoride (PVDF) membranes were purchased from Millipore (Bedford, MA, USA). The primary and secondary antibodies including Bax (#2772), Bcl-2 (ab196495), SOD (ab13498), CAT (#14097), GPx (ab22604), anti-rabbit IgG, HRP-linked antibody (#7074), anti-mouse IgG, HRP-linked antibody (#7076), and beta-actin (#8457) were purchased from Cell Signaling Technology (Beverly, MA, USA) and Abcam (Cambridge, UK).

### 2.2. DPPH Radical Scavenging Activity

The scavenging capacity of the extracts against DPPH was measured according to the method of Hatano et al. [18]. Briefly, in a 96-well plate, a solution of 60 µM DPPH was mixed with 100 µL of the extracts prepared at 50, 100, 250, and 500 µg/mL. The mixture was left in the dark for 30 min at room temperature, and then the absorbance was measured at 540 nm using a microplate reader (Rayto Life and Analytical Sciences Co., Ltd., Shenzhen, China). The DPPH radical scavenging activity (%) compared to the control was expressed as follows.

DPPH scavenging activity (%) = [(Abs_control_ − Abs_sample_)/Abs_control_] × 100

Abs_control_ = The absorbance of the DPPH radical in methanol.

Abs_sample_ = The absorbance of the DPPH radical solution with CTS and TC.

### 2.3. Hydroxyl Radical (·OH) Scavenging Activity

The method described by Gutteridge [19] was used to measure the ·OH scavenging capacity of the extracts. Briefly, 10 mM FeSO_4_·7H_2_O-EDTA, 10 mM 2-deoxyribose, and 10 mM H_2_O_2_ were added to each extract. The mixture was then incubated at 37 °C while being shielded from light. After 4 h, a 1% TBA solution and a 2.8% TCA solution were added to the mixture and left in a water bath (100 °C) for 20 min. The absorbance was measured at 490 nm, and the ·OH radical scavenging activity (%) compared to the control was calculated as follows.

OH radical scavenging activity (%) = [(Abs_control_ − Abs_sample_)/Abs_control_] × 100

Abs_control_ = The absorbance of the ·OH radical in distilled water.

Abs_sample_ = The absorbance of the ·OH radical solution with CTS and TC.

### 2.4. Culture and Treatment of Cells

CRL-2266, a human neuroblastoma SH-SY5Y cell line, was purchased from the American Type Culture Collection (Manassas, VA, USA). In a 5% CO_2_ incubator at 37 °C, cells were grown in DMEM supplemented with 10% FBS and 1% penicillin-streptomycin. The cells were then seeded on 96-well plates or 6-well plates at densities of 5 × 10^4^ or 1 × 10^6^ cells/mL for 24 h. Cells were pretreated with CTS, TC, and their combination (10 µg/mL) for 4 h and then added with H_2_O_2_ (300 µM) for another 24 h.

### 2.5. Cell Viability

The viability of the cells was assessed using the MTT colorimetric test. A solution of 5 mg/mL MTT was added to each well and incubated for 4 h at 37 °C. Following the removal of the supernatant, DMSO was added in order to dissolve the formazan crystals. With the use of a microplate reader, the absorbance was determined at 540 nm.

### 2.6. Hoechst 33,342 Staining

Cells were seeded on 8-well chamber slides, pre-treated with the extracts for 4 h, and added with H_2_O_2_ for 24 h. Then, the cells were fixed with 4% paraformaldehyde for 10 min and stained with a Hoechst 33,342 solution (10 µg/mL) for 20 min. The nuclear morphology was observed using a fluorescence microscope (Olympus BX50, Tokyo, Japan). In untreated cells, the nuclei appear round. In apoptotic conditions, the cell bodies are fragmented [20].

### 2.7. LDH Release

An LDH cytotoxicity detection kit (#MK402, TaKaRa Bio Inc., Otsu, Japan) was used to measure LDH release, in line with the manufacturer’s instructions. Briefly, equal parts of the cell supernatant and the LDH solution were mixed, and the mixture was then left to sit at room temperature in the dark for 30 min. The absorbance at 490 nm was recorded.

### 2.8. ROS Production

The formation of intracellular ROS was measured using the DCF-DA fluorescence assay. After removing the cell supernatant, DCF-DA (80 µM) was added to the cells. Cells were then incubated at 37 °C for 30 min. The fluorescence intensity was measured at an excitation wavelength of 480 nm and an emission wavelength of 535 nm using a fluorescence spectrophotometer (BMG Labtech., Ortenberg, Germany).

### 2.9. NO Generation

Cell supernatant was mixed with an equal amount of Griess reagent and left at room temperature for 30 min while being shielded from light in order to determine the NO level. The absorbance was recorded at 540 nm.

### 2.10. Western Blotting

Cells were harvested and lysed using a lysis buffer (a mixture of RIPA buffer and protease inhibitor cocktail). Equal amounts of proteins were electrophoretically separated by 10% or 13% SDS-polyacrylamide gel and transferred to PVDF membranes. The membranes were blocked in 5% skim milk for 1 h. The membranes were incubated with primary antibodies (Bax, 1:1000; Bcl-2, 1:500; SOD, 1:5000; CAT, 1:1000; GPx, 1:1000; beta-actin, 1:1000) and then HRP-conjugated secondary antibodies. Protein bands were visualized by a chemiluminescence imaging system (Davinch-Chemi^TM^, CoreBio, Seoul, Republic of Korea), according to the manufacturer’s instructions.

### 2.11. Statistic Evaluation

The mean ± standard deviation (SD) for all the data was displayed. Experiments were replicated in triplicate. Data analysis was carried out utilizing SPSS 23.0 (IBM). For the examination of significant differences, a one-way ANOVA was utilized in conjunction with Duncan’s multiple test. Statistics were considered significant for *p*-values under 0.05.

## 3. Results

### 3.1. Effects of CTS, TC, and Their Combination on Free Radical Scavenging Activities

We investigated in vitro DPPH and ·OH radical scavenging activities of CTS, TC, and CTS + TC at 50, 100, 250, and 500 µg/mL. As shown in Table 1, DPPH radical scavenging activity dose-dependently increased by CTS, TC, and CTS + TC. The percentage of inhibition by CTS + TC showed 11.61%, 38.21%, and 78.36%, and 85.67% at 50, 100, 250, and 500 µg/mL, which was higher than CTS or TC at all concentrations. Moreover, the IC_50_ values of CTS, TC, and CTS + TC were 298.41 µg/mL, 222.67 µg/mL, and 140.09 µg/mL, respectively The DPPH radical scavenging capacity of the extracts is in the order as follows: CTS + TC > TC > CTS.

As shown in Table 2, the ·OH radical scavenging activity of CTS, TC, and their combination increased dose-dependently. The scavenging abilities of CTS + TC at all concentrations were 54.72%, 55.87%, 64.99%, and 78.87%. The highest ·OH radical scavenging capacity was shown in the group of CTS + TC at 500 µg/mL compared to CTS or TC alone. IC_50_ values of CTS, TC, and CTS + TC were 401.3 µg/mL, 167.3 µg/mL, and 44.62 µg/mL. The ·OH radical scavenging capacity increased in the order CTS + TC > TC > CTS.

### 3.2. Effects of CTS, TC, and Their Combination on Neuronal Cell Damage in H_2_O_2_-Stimulated SH-SY5Y Cells

**Figure 1:**
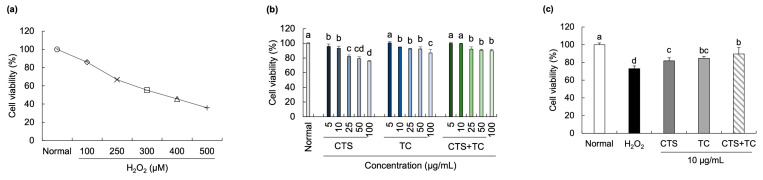
Effect of *Carthamus tinctorius* L. seed, *Taraxacum coreanum*, and their combination on H_2_O_2_-induced neurotoxicity in SH-SY5Y cells. Cell viability of H_2_O_2_ (**a**), extracts (**b**), and extracts with H_2_O_2_ (**c**). The values are mean ± SD. According to Duncan’s multiple range test, means with different letters (a–d) are significantly different (*p* < 0.05). CTS, *C. tinctorius* L. seed; TC, *T. coreanum*; CTS + TC, a combination of CTS and TC.

We investigated the protective effects of CTS, TC, and their combination against SH-SY5Y cell damage induced by H_2_O_2_. The cells were exposed to H_2_O_2_ at concentrations ranging from 100 µM to 500 µM, which exhibited an approximately 55% cell survival rate at 300 µM (Figure 1a). Various concentrations (5, 10, 25, 50, 100 µg/mL) of CTS, TC, and CTS + TC were treated in the cells, showing significantly decreased cell viability from 25 µg/mL to 100 µg/mL compared with the normal cells (Figure 1b). Therefore, we examined 10 µg/mL of CTS, TC, and their combination to evaluate the protective effects against H_2_O_2_ (300 µM)-induced cytotoxicity in SH-SY5Y cells. In Figure 1c, when the cells were exposed to H_2_O_2_, the cell viability was significantly reduced compared with the normal cells. However, decreased cell viability was recovered by the pretreatment with CTS, TC, and CTS + TC, respectively. CTS + TC showed a higher cell survival rate (89.52%) compared with CTS or TC alone.

**Figure 2:**
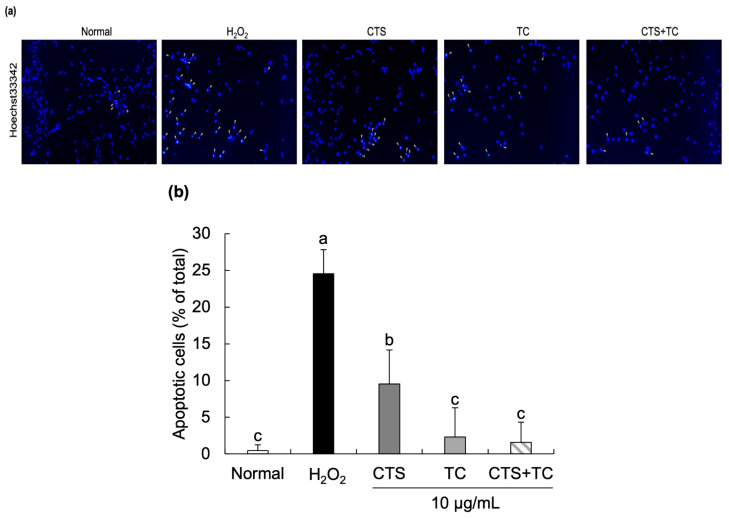
Effect of *Carthamus tinctorius* L. seed, *Taraxacum coreanum*, and their combination on morphological changes in H_2_O_2_-induced SH-SY5Y cells. Nuclear morphological changes of the cells after Hoechst 33,342 staining (**a**) and quantification analysis of apoptotic cells (**b**). The values are mean ± SD. According to Duncan’s multiple range test, means with different letters (a–c) are significantly different (*p* < 0.05). CTS, *C. tinctorius* L. seed; TC, *T. coreanum*; CTS + TC, a combination of CTS and TC.

The morphological changes in H_2_O_2_-treated cells were imaged by a fluorescence microscope (Figure 2). Compared with the normal cells, significant cell number reduction and apoptotic bodies were observed after exposure to H_2_O_2_. In contrast, fewer apoptotic cells were observed in the cells with the addition of CTS, TC, and their combination. Particularly, CTS + TC showed fewer changes in nuclei morphology than CTS or TC alone, suggesting that the combination might have a synergistic protective effect on apoptotic cells.

**Figure 3:**
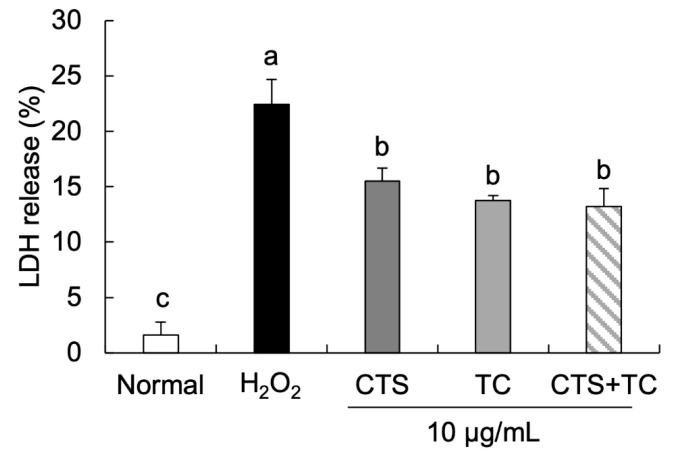
Effect of *Carthamus tinctorius* L. seed, *Taraxacum coreanum*, and their combination on LDH release in H_2_O_2_-induced SH-SY5Y cells. Values are mean ± SD. According to Duncan’s multiple range test, means with different letters (a–c) are significantly different (*p* < 0.05). CTS, *C. tinctorius* L. seed; TC, *T. coreanum*; CTS + TC, a combination of CTS and TC.

A low level of LDH release was shown in the normal cells compared with H_2_O_2_-treated cells (Figure 3). However, treatment with CTS (15.50%), TC (13.75%), and their combination (13.21%) significantly inhibited the release of LDH compared with the control cells (22.46%).

### 3.3. Effects of CTS, TC, and Their Combination on ROS Production in H_2_O_2_-Stimulated SH-SY5Y Cells

**Figure 4:**
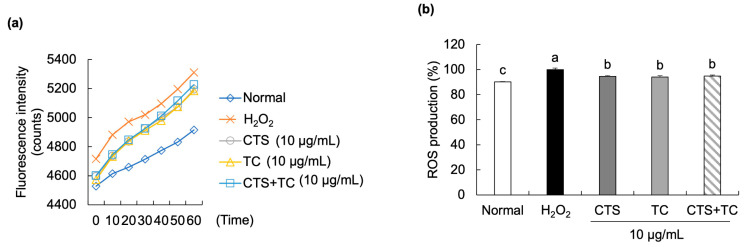
Effect of *Carthamus tinctorius* L. seed, *Taraxacum coreanum*, and their combination on ROS generation in H_2_O_2_-stimulated SH-SY5Y cells. Fluorescence intensity during 60 min (**a**), and ROS production at 60 min (**b**). Values are mean ± SD. According to Duncan’s multiple range test, means with different letters (a–c) are substantially different (*p* < 0.05). CTS, *C. tinctorius* L. seed; TC, *T. coreanum*; CTS + TC, a combination of CTS and TC.

As shown in Figure 4a, the fluorescence intensity in the H_2_O_2_-treated control group rapidly increased during 60 min in a time-dependent manner. However, the extracts of CTS, TC, and their combination significantly slowed the increased rate of ROS production in H_2_O_2_-treated SH-SY5Y cells. Further, at 60 min (Figure 4b), the percentage of ROS production significantly increased in the H_2_O_2_-treated control cells (set as 100%) compared to the normal cells (90.13%) and remarkedly reduced by the treatment of CTS (94.56%), TC (94.00%), and their combination (94.85%).

### 3.4. Effects of CTS, TC, and Their Combination on NO Generation in H_2_O_2_-Stimulated SH-SY5Y Cells

**Figure 5:**
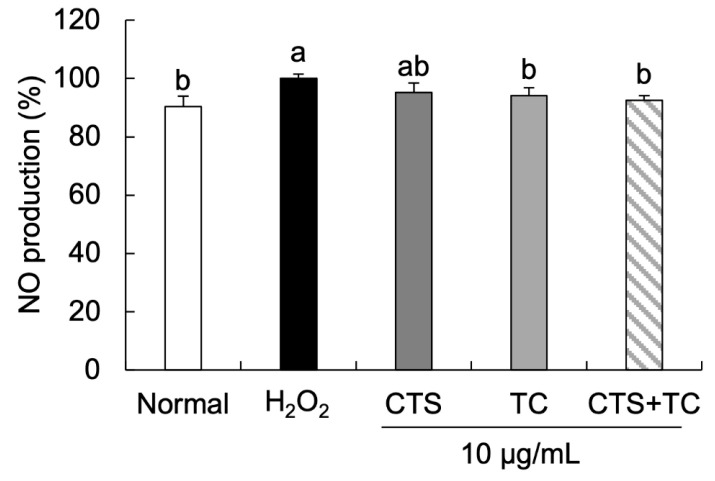
Effect of *Carthamus tinctorius* L. seed, *Taraxacum coreanum*, and their combination on NO production in H_2_O_2_-induced SH-SY5Y cells. The values are mean ± SD. According to Duncan’s multiple range test, means with different letters (a,b) are significantly different (*p* < 0.05). CTS, *C. tinctorius* L. seed; TC, *T. coreanum*; CTS + TC, a combination of CTS and TC.

As shown in Figure 5, H_2_O_2_ markedly increased NO production (set as 100%), which was significantly recovered by the treatment of TC and CTS + TC, showing 94.19% and 92.47%, respectively. These findings suggest that CTS + TC exerts a higher protective effect against H_2_O_2_-induced neuronal oxidative stress than CTS or TC alone in SH-SY5Y cells.

### 3.5. Effects of CTS, TC, and Their Combination on Protein Expression of Antioxidant Enzymes in H_2_O_2_-Treated SH-SY5Y Cells

**Figure 6:**
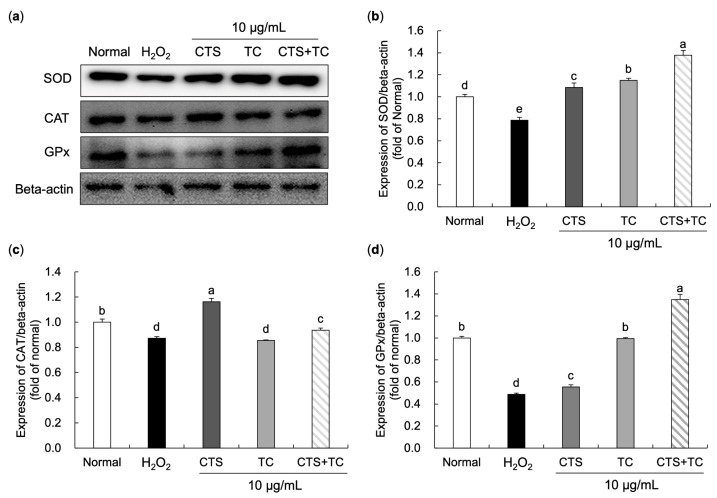
Effect of *Carthamus tinctorius* L. seed, *Taraxacum coreanum*, and their combination on protein expression of antioxidative enzymes in H_2_O_2_-treated SH-SY5Y cells. The western blot bands (**a**), relative protein levels of SOD (**b**), CAT (**c**), and GPx (**d**). The values are mean ± SD. According to Duncan’s multiple range test, means with different letters (a–e) are significantly different (*p* < 0.05). Beta-actin was used as a loading control. CTS, *C. tinctorius* L. seed; TC, *T. coreanum*; CTS + TC, a combination of CTS and TC; SOD, superoxide dismutase; CAT, catalase; GPx, glutathione peroxidase.

As shown in Figure 6, protein expressions of SOD (78.79%), CAT (87.28%), and GPx (48.73%) significantly decreased H_2_O_2_ compared to the normal group. However, the expressions of these proteins were increased by the treatment of CTS, TC, and their combination. We found that up-regulated protein expressions of SOD and GPx in H_2_O_2_-induced SH-SY5Y cells were markedly shown in the CTS (108.47% and 55.51%, respectively), TC (114.93% and 99.50%, respectively), and CTS + TC (137.68% and 134.82%, respectively) groups. The expression of CAT was significantly up-regulated by CTS and CTS + TC, but there was no significant change in the TC group.

### 3.6. Effects of CTS, TC, and Their Combination on Bax and Bcl-2 Protein Expressions in H_2_O_2_-Stimulated SH-SY5Y Cells

**Figure 7:**
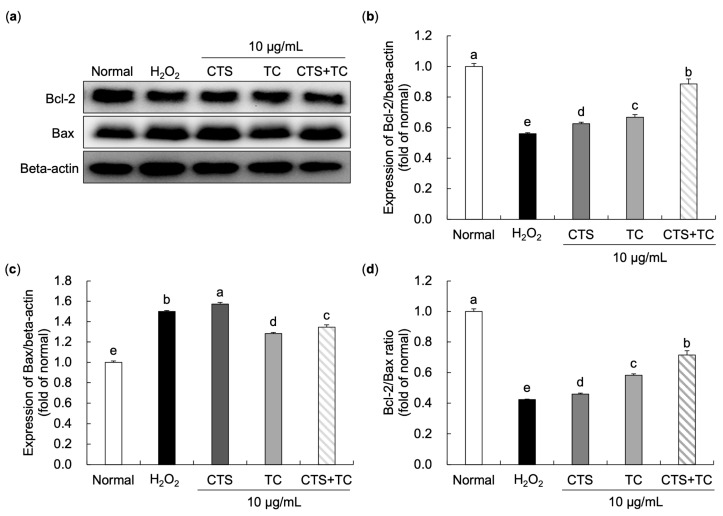
Effect of *Carthamus tinctorius* L. seed, *Taraxacum coreanum*, and their combination on anti-apoptotic activity in H_2_O_2_-stimulated SH-SY5Y cells. The western blot bands (**a**), relative protein levels of Bcl-2 (**b**), Bax (**c**), and Bcl-2/Bax ratio (**d**). The values are mean ± SD. According to Duncan’s multiple range test, means with different letters (a–e) are significantly different (*p* < 0.05). Beta-actin was used as a loading control. CTS, *C. tinctorius* L. seed; TC, *T. coreanum*; CTS + TC, a combination of CTS and TC; Bcl-2, B-cell lymphoma 2; Bax, B-cell lymphoma 2 associated X protein.

The protein expressions of Bcl-2 and Bax were measured (Figure 7). The result showed a 1.50-fold increase in Bax level (149.94%) and a decrease in Bcl-2 level (56.02%) in SH-SY5Y cells exposed to H_2_O_2_, indicating that cell apoptosis could be induced by H_2_O_2_. However, treating with the combination of CTS and TC significantly down-regulated Bax expression (134.52%) and up-regulated Bcl-2 expression (88.65%). Moreover, the Bcl-2 to Bax showed a significantly reduced ratio (42.36%) in the H_2_O_2_-treated control group but recovered in CTS + TC (71.55%). The result suggests that CTS + TC has a greater protective ability on H_2_O_2_-induced apoptosis.

## 4. Discussion

In oxidative stress-mediated disorders, the synergism of combination has been found to provide better therapeutic effects. The co-administration of L-carnosine and (-)-epigallocatechin-3-gallate has been demonstrated to show a synergistic effect against neuronal damage induced by oxidative stress via the regulation of the heat shock protein 72/HO-1 pathway [21]. The co-treatment of *Vitis vinifera* L. leaf and *Centella asiatica* showed a significantly decreased NO level and intracellular ROS generation in H_2_O_2_-treated endothelial cells than *V. vinifera* L. leaf or *C. asiatica* [22].

CTS is widely used as a raw material in edible oil and has high nutritional value, containing approximately 70% linoleic acid [7]. Moreover, phenolic compounds including *N*-feruloyl serotonin, *N*-(*p*-coumaroyl) serotonin, luteolin, and acacetin have been isolated from the ethyl acetate fraction of the CTS extracts [7,23]. In particular, *N*-feruloyl serotonin and *N*-(*p*-coumaroyl) serotonin, which are known as serotonin derivatives in CTS, have been reported to represent 58% and 34% of the total phenolic compound [23]. TC is considered a non-toxic herb, and the whole plant can be used for medicine due to the varied phytochemicals such as sesquiterpene lactones, phenylpropanoids, and terpenoids contained from the flower to the root [10]. In H_2_O_2_-stimulated C6 glioma cells, luteolin and luteolin-7-glucoside from the ethyl acetate fraction of TC have been shown to diminish oxidative stress, indicating a protective effect in neuronal cells by inhibiting ROS production [24]. Compared with CTS or TC alone, in vivo studies have shown that the CTS and TC combination has synergistic neuroprotective benefits in amyloid beta-injected AD mouse models [25]. However, the antioxidant and neuroprotective effects of CTS and TC combination under cellular systems have yet to be studied; thus, this study demonstrated an effect on antioxidative stress by the combination in H_2_O_2_-treated SH-SY5Y cells. Therefore, this study investigated the neuroprotective effects and mechanisms of a combination of CTS and TC in oxidative stress-stimulated SH-SY5Y cells treated by H_2_O_2_.

The HPLC analysis of CTS, TC, and their combination has been carried out in the preliminary experiment. Five substances, including serotonin, chlorogenic acid, chicoric acid, *N*-(*p*-coumaroyl) serotonin, and *N*-feruloyl serotonin, were presented in the combination of CTS and TC. It has been reported that CTS is the primary source of *N*-(p-coumaroyl) serotonin and *N*-feruloyl serotonin, while TC contains chicoric acid and chlorogenic acid. In our initial research, the combination contained high levels of chicoric acid and *N*-feruloyl serotonin. Further, we explored the neuroprotective and antioxidative effects of these two compounds, respectively, and the results showed their activities against neurodegeneration and oxidative stress [26].

The overproduction of free radicals is involved in oxidative stress [27]. DPPH is a dark-colored and stable N-atom-centered free-radical molecule, which is frequently used for the measurement of the antioxidant ability. Chemicals react with DPPH by H-atom donation or electron transfer, changing the color into pale yellow, which can be detected using spectrophotometers and thus predict the antioxidant activities of various compounds or extracts [28]. ·OH is known to be the most biologically active free radical that causes DNA damage, induces neurotoxicity, and ultimately implicates the occurrence of neurodegenerative diseases [29]. In the previous studies, DPPH and hydroxyl radical scavenging activities of CTS ethanol extract at concentrations of 5 to 500 µg/mL have been measured, and the IC_50_ value was at about 100 and 6 µg/mL, respectively [12]. Moreover, the chloroform fraction of TC treated to macrophages from mice showed no toxic effect up to 200 µg/mL [30]. Based on these studies, consistent concentrations at 50, 100, 250, and 500 µg/mL were used to measure the in vitro antioxidant assays in this study. CTS, TC, and their combination exhibited DPPH and ·OH radical scavenging capacities in a dose-dependent manner. The ethanolic extract of CTS has revealed effects against DPPH, ·OH, singlet oxide, and nitric oxide radical scavenging, indicating an antioxidative property of CTS in vitro [12]. Moreover, the radical scavenging activity against DPPH by different parts of TC has been measured, showing IC_50_ values at 232.40 µg/mL in the aerial part and 693.64 µg/mL in the root [31]. In particular, we found that CTS + TC has a greater free radical scavenging activity against DPPH and ·OH free radicals in comparison to CTS or TC alone. These results indicated that the CTS and TC combination could play a synergy effect from the free radical production.

The MTT assay for measuring the viable cells is based on mitochondrial activity, which is linearly related to the number of viable cells [32]. It has been reported that apoptotic cells undergo morphological changes, including nuclear condensation and DNA fragmentation [33]. Hoechst staining is a method used for nuclear staining in living and fixed cells to observe morphological changes of the cells [34]. In our results, the cytotoxicity of each sample was measured. The concentration of CTS showed significantly decreased cell viability from 25 µg/mL compared with the control group, even though TC and the combination (CTS + TC) were safe at higher doses; as a result, the concentration of CTS, TC, and their combination was determined at 10 µg/mL for use in this study. Furthermore, compared to the normal cells, the addition of H_2_O_2_ induced cell damage; however, this damage was reversed by the treatment of CTS, TC, and their combination. In particular, the CTS and TC combination synergistically increased the cell viability more than the single treatment. The Hoechst33342 staining test further supported these findings, which showed significant apoptotic bodies and reduced cell numbers in the H_2_O_2_-treated group, whereas they were effectively reversed by the combination of CTS and TC. While cell membranes are being destroyed, LDH, a stable cytoplasmic enzyme, can be quickly released into the cell supernatant [35]. It has been demonstrated that cytotoxicity is significantly induced by H_2_O_2_ treatment by increasing LDH release in PC12 neuronal cells [36]. In the present study, treatment of H_2_O_2_ increased LDH levels in SH-SY5Y cells, which indicated a release out of the cell membrane and an effect on cellular toxicity; however, the treatment of CTS, TC, and their combination markedly reduced LDH release.

Oxidative stress due to the excessive production of ROS induced by H_2_O_2_ can cause neuronal cell damage, resulting in cell death [37]. To detect the generation of ROS, a DCF-DA assay is one of the widely used methods. A DCF-DA reagent can be used to follow the changes in ROS over time [38]. ROS generation has efficiently been induced by H_2_O_2_. The exposure of SH-SY5Y cells to H_2_O_2_ has been demonstrated to increase ROS production by fluorescence imaging [39]. It has been reported that neuronal damage induced by the increased generation of ROS can activate cellular stress pathways [40]. Choi et al. demonstrated that CTS can significantly reduce ethanol-increased ROS levels in C6 glial cells [12]. Luteolin and luteolin-7-glucoside, flavonoids from TC, have been reported to decrease ROS production in LPS/IFN-γ-treated RAW 264.7 cells [41]. In our results, H_2_O_2_-induced cellular ROS production mediates oxidative stress but could be recovered by the treatment of CTS and TC combination, suggesting that the combination may exhibit an antioxidative effect by ROS scavenging activity.

NO affects cell death by inhibiting cytochrome oxidase and mitochondrial respiration, resulting in the leakage of H_2_O_2_ production from mitochondria. High levels of NO cause oxidative damage to cells and induce apoptosis [42]. Hussain et al. have demonstrated that the NO level was significantly increased in H_2_O_2_-treated lymphocytes [43]. In the present study, the increased NO production induced by H_2_O_2_ was suppressed by the treatment of TC and the combination but not the treatment of CTS, suggesting that the CTS and TC combination might show a protective effect on the production of NO.

A previous study has reported that the accumulation of ROS leads to the depletion of antioxidants and attacks proteins, lipids, and DNA [44]. SOD, CAT, and GPx are antioxidant enzymes that increase their activities under oxidative stress conditions to prevent the generation of free radicals in cells, acting as the first line of defense antioxidants [45]. Generally, SOD converts superoxide into H_2_O_2_ and oxygen, and H_2_O_2_ further breaks down into water and oxygen by CAT and GPx. In the present study, H_2_O_2_ was used to induce oxidative damage since the extremely toxic ·OH radical can be easily and rapidly generated through the Fenton reaction with the presence of Fe^2+^ [46]. It resulted in cell damage by down-regulating the SOD, CAT, and GPx protein expressions in the H_2_O_2_-treated group. It has been reported that the extract of CTS increases the levels of SOD, GPx, and CAT in high fructose-treated rats, indicating that CTS could improve oxidative injury [47]. Moreover, TC has been shown higher SOD activity than the general dandelion *T. officinale* [48], and it markedly increased the activities of SOD and CAT in *N*-nitrosodiethylamine-induced liver injury [49]. In this study, CTS and TC combination showed an enhanced effect on the SOD and GPx protein expressions compared to CTS or TC alone, suggesting that the combination might act as a synergistically protective effect to defend against oxidative stress by restoring antioxidant enzyme activities.

Apoptosis is regulated by many genes, among which the Bcl-2 family is the most prevalent. Apoptosis inducer (Bax) and inhibitor (Bcl-2) proteins make up the protein superfamily known as Bcl-2. The overexpression of Bax accelerates the apoptotic death, whereas Bcl-2 acts an opposite function. It has been reported that the balance between Bax and Bcl-2 is important in maintaining cell morphology and function [50]. Additionally, it has been suggested that the Bax/Bcl-2 ratio may serve as a marker for the susceptibility of a cell to undergo apoptosis [51]. The decrease in the Bcl-2/Bax protein ratio has been demonstrated to be associated with the induction of H_2_O_2_ [52]. According to a previous study, CTS treatment of mice greatly down-regulated Bax expression responsible for kidney damage caused by cisplatin [53]. In the present study, a highly increased ratio of Bcl-2 to Bax was observed in the normal group compared to the H_2_O_2_-treated group, confirming the cell damage by H_2_O_2_. The result was recovered by the treatment of CTS, TC, and their combination; particularly, the combination performed a synergy effect, suggesting a protective ability of CTS and TC combination on cell apoptosis.

In the present study, we explored the antioxidative stress effect of the combination (CTS + TC) compared with CTS or TC. The results of free radical scavenging activity were significant and showed a synergistic effect of the combination compared to the single herbs. In the results from cellular experiments, despite the fact that the percentage of LDH release and ROS production did not show significant differences among CTS, TC, and the combination, there were differences in numerical values. The apoptotic cells and NO production, as well the protein expression of Bcl-2 and Bax, seemed to show better effects on the combination, suggesting its potential effect against inflammation and apoptosis. Similarly, in the expression of SOD and GPx, the combination exhibits a greater antioxidative effect than CTS or TC alone. This study provided neuroprotective effects of the combination of CTS and TC on neuronal oxidative stress induced by H_2_O_2_. However, there are limitations in the present study, and more details about the combination of antioxidative stress through other mechanisms, such as NF-ĸB and Nrf2/HO-1 signaling pathways, need further investigation.

## 5. Conclusions

Taken together, the CTS and TC combination had higher free radical scavenging activities than CTS or TC alone. In addition, the combination offered a synergistic effect on preventing H_2_O_2_-stimulated cell damage in SH-SY5Y cells by inhibiting LDH release, NO production, and ROS generation. Moreover, the combination regulated apoptotic responses in cells by regulating the Bcl-2/Bax ratio and up-regulating the antioxidative protein expressions of SOD, CAT, and GPx. These findings suggest that CTS and TC combination can be used to prevent and treat oxidative stress-related neurodegenerative diseases.

## Figures and Tables

**Table 1:** DPPH radical scavenging activities of the seeds *Carthamus tinctorius* L. seed, *Taraxacum coreanum*, and their combination.

Concentration (µg/mL)	DPPH Radical Scavenging Activity (%)		
	CTS	TC	CTS + TC
50	2.43 ± 4.34 ^c^	10.31 ± 4.28 ^c^	11.61 ± 3.44 ^d^
100	13.83 ± 3.27 ^b^	20.73 ± 3.17 ^b^	38.21 ± 3.42 ^c^
250	55.17 ± 10.88 ^a^	63.31 ± 5.71 ^a^	78.36 ± 5.50 ^b^
500	60.29 ± 7.35 ^a^	64.97 ± 4.65 ^a^	86.67 ± 5.92 ^a^
IC_50_ (µg/mL)	302.63	239.80	138.34

Values are mean ± SD. ^a–d^ Means with the different letters are significantly different (*p* < 0.05) according to Duncan’s multiple range test. CTS, *C. tinctorius* L. seed; TC, *T. coreanum*; CTS + TC, a combination of CTS and TC; IC_50_, half-maximal inhibitory concentration.

**Table 2:** ∙OH radical scavenging activities of the *Carthamus tinctorius* L. seed, *Taraxacum coreanum*, and their combination.

Concentration (µg/mL)	OH Radical Scavenging Activity (%)		
	CTS	TC	CTS + TC
50	39.41 ± 1.27 ^c^	41.41 ± 3.67 ^c^	54.72 ± 0.71 ^c^
100	43.38 ± 3.31 ^bc^	46.86 ± 4.16 ^b^	55.87 ± 4.63 ^c^
250	45.81 ± 1.86 ^b^	51.67 ± 0.96 ^b^	64.99 ± 1.81 ^b^
500	52.19 ± 4.28 ^a^	58.46 ± 3.12 ^a^	78.87 ± 3.95 ^a^
IC_50_ (µg/mL)	400.66	167.34	44.73

Values are mean ± SD. According to Duncan’s multiple range test, means with different letters (^a–c^) are substantially different (*p* < 0.05). CTS, *C. tinctorius* L. seed; TC, *T. coreanum*; CTS + TC, a combination of CTS and TC; IC_50_, half-maximal inhibitory concentration.

## Data Availability

The data used to support the findings of this study can be made available by the corresponding author upon request.
